# Alkaline Stable Anion Exchange Membranes Based on Cross-Linked Poly(arylene ether sulfone) Bearing Dual Quaternary Piperidines for Enhanced Anion Conductivity at Low Water Uptake

**DOI:** 10.3390/molecules27020364

**Published:** 2022-01-07

**Authors:** Prem P. Sharma, Yeeun Jeon, Dukjoon Kim

**Affiliations:** School of Chemical Engineering, Sungkyunkwan University, Suwon 440-746, Gyeonggi, Korea; premsharma15@gmail.com (P.P.S.); jyeun00513@gmail.com (Y.J.)

**Keywords:** anion exchange membrane, ionic conductivity, poly(arylene ether sulfone), alkaline stability, crosslink, fuel cell, low water uptake, quaternary piperidinium

## Abstract

Alkaline stable anion exchange membranes based on the cross-linked poly(arylene ether sulfone) grafted with dual quaternary piperidine (XPAES-DP) units were synthesized. The chemical structure of the synthesized PAES-DP was validated using ^1^H-NMR and FT-IR spectroscopy. The physicochemical, thermal, and mechanical properties of XPAES-DP membranes were compared with those of two linear PAES based membranes grafted with single piperidine (PAES-P) unit and conventional trimethyl amine (PAES-TM). XPAES-DP membrane showed the ionic conductivity of 0.021 S cm^−1^ at 40 °C which was much higher than that of PAES-P and PAES-TM because of the possession of more quaternary ammonium groups in the cross-linked structure. This cross-linked structure of the XPAES-DP membrane resulted in a higher tensile strength of 18.11 MPa than that of PAES-P, 17.09 MPa. In addition, as the XPAES-DP membrane shows consistency in the ionic conductivity even after 96 h in 3 M KOH solution with a minor change, its chemical stability was assured for the application of anion exchange membrane fuel cell. The single-cell assembled with XPAES-DP membrane displayed a power density of 109 mWcm^−2^ at 80 °C under 100% relative humidity.

## 1. Introduction

Recently, the proton exchange membrane fuel cell (PEMFC) is an emerging eco-friendly technology to satisfy the demand for electrical power. However, because of utilization of the expensive platinum catalysts and perfluoro-sulfonate ionomers, its commercialization still has been limited. The demand for more facile technologies which can compete PEMFCs has led to huge attention to the development of the anion exchange membrane fuel cell (AEMFC) [1,2,3,4,5,6]. The AEMFC offers relatively mild operations under high pH environments and broad selection of reactant species. Additionally, it provides a platform for non-noble catalysts such as Ni and Ag, because of its faster oxygen reduction kinetics to sustain its competency with a comparison of other technologies for energy application [7,8,9,10,11,12]. Despite all these advantages, its wide application has been still been restricted due to the drawbacks of anionic exchange polymer electrolyte membranes and the low anion conductivity with low alkaline stability [13,14,15,16,17].

To mitigate these weaknesses, several strategies have been addressed by designing polymer structures functionalized with different cationic groups such as metal-centered cations, phosphonium, and quaternary ammonium groups [18,19]. Quaternary ammonium is one of the most widely studied functional groups because of its ease of raw material acquisition, synthetic protocols, and high anionic conductivity [20,21]. However, the attack of nucleophiles in the form of hydroxide ions on quaternary ammonium group is the main obstacle in its application, as it de-bonds the functional groups, followed by the subsequent degradation of polymer backbone by a variety of mechanisms depending on its structure [22,23]. Thus, the designing of the macromolecular structures attached with suitable types of quaternary ammonium groups is of quite general importance.

Many works of literature have revealed several strategies to enhance the alkaline stability of anion exchange polymer membranes. Grafting long alkyl chains (propyl, butyl, etc.) to the main polymer backbone is one of the great strategies that builds up a distance, which in turn avoids the invasion of nucleophiles [24]. Employment of quaternary ammonium groups by conventional methods usually results in the formation of polymer structures directly linked with them. The quaternary ammonium groups which are directly tethered to aromatic polymer backbones are proven to be quite sensitive towards nucleophilic attack by OH^−^, and thus responsible for initiating cleavage of polymer backbones in alkali media [25]. Among a huge number of cationic groups including imidazolium, guanidinium, and phosphonium, cycloaliphatic quaternary ammonium groups are reported to exhibit exceptionally high stability against nucleophiles because they contain β-protons with the –C–C– bond which rotationally protect the ring geometry [26,27]. On the other hand, an increment in the number of carbon atoms (>4) in alkyl groups, provides the steric hindrance effect which in turn increases the energy barrier against Hoffman elimination, a major cause for polymer degradation.

In this work, the cross-linked poly(ether arylene sulfone) (XPAES) with the dual quaternized functional groups has been synthesized to be compared with the two linear PAES with the different quaternized groups, investigating various physicochemical, thermal, mechanical, and chemical stabilities. The presence of dual quaternized groups will promote the anion conductivity of the prepared anion exchange membrane. On the other hand, as the polycyclic ring works as a steric hindrance to the nucleophile, this bicyclic ring plays a role against its deformation. Additionally, the cross-linked structure would provide mechanical strength with the polymer membrane which eventually enhances its alkaline stability.

## 2. Results and Discussion

### 2.1. Chemical Structure Characterization

**Scheme 1:**
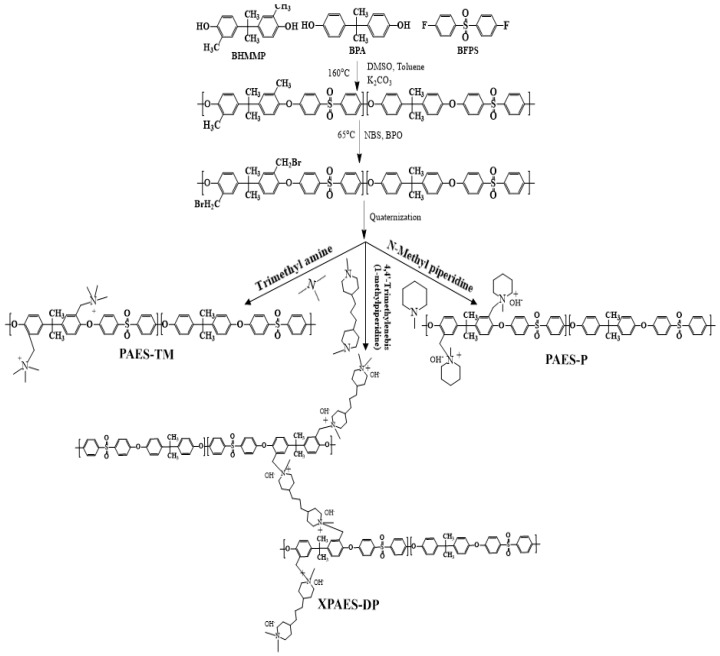
Reaction scheme for the synthesis of XPAES-DP, PAES-P and PAES-TM.

**Figure 1:**
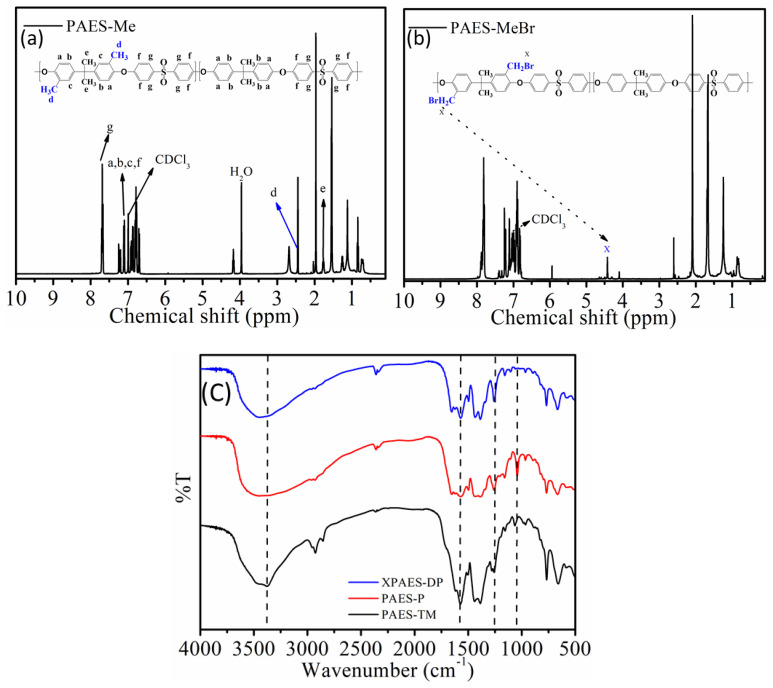
^1^H NMR spectra of (**a**) PAES-Me and (**b**) PAES-MeBr to confirm the stepwise functionalization of PAES, and (**c**) FT-IR spectra of three synthesized membranes, XPAES-DP, PAES-P, and PAES-TM.

The synthetic scheme of the polymers are shown in Scheme 1 and their chemical structure was characterized by ^1^H-NMR spectroscopy as shown Figure 1a,b, respectively. In this first step, the grafting of methylated groups directly takes place at the aromatic ring of PAES backbone. The chemical shifts associated with the protons in PAES backbone are indicated by two distinct signals at 1.8 and 2.06 ppm. Moreover, the broad and multiple signals in the region 6–8 ppm are due to the protons of the methyl group attached to the aromatic rings in Figure 1a. In the second step, as the signal appeared at 4.41 ppm arises from the attachment of -MeBr group to the PAES backbone in Figure 1b, it confirms the conversion of methylated group into bromomethylated group. Figure 1c represents the FT-IR spectra of the synthesized membranes. The stretching vibration at 1021 cm^−1^ is from the C–O starching associated with aryl alkyl ether which is consistent in all three membranes. A distinct IR band at 1540 cm^−1^ arises from the stretching vibration of –C–N bond of the ammonium group which is more dominant in XPAES-DP, supporting the presence of more quaternary ammonium groups associated with cross-linked structure. Moreover, a broad IR band in the region 2930 cm^−1^ is associated with –CH stretching vibration, while those in the region nearby 1640 cm^−1^ is correlated with the vibration of –C=C– present in the aromatic ring.

The ion exchange capacity (IEC) was measured by titration method. XPAES-DP showed the highest IEC of 1.04 meq g^−1^ because of more functional groups. That of PAES-P and PAES-TM was 0.91 and 0.88 meq g^−1^, respectively.

### 2.2. Water Uptake and Swelling Ratio

**Figure 2:**
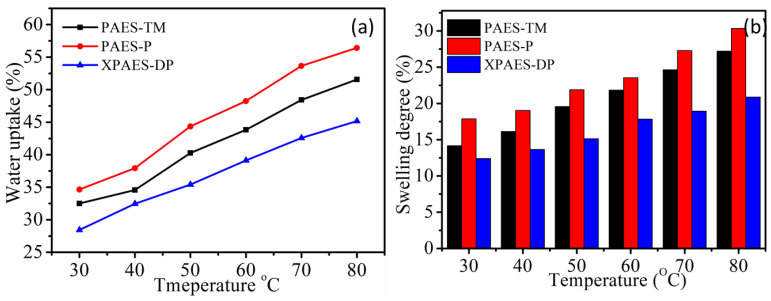
(**a**) Water uptake and (**b**) swelling ratio of different membranes at different temperature.

Water uptake and swelling ratio are important properties of the ion exchange membranes, as they significantly affect other membrane properties. In general, high water uptake enhances the ion conductivity but reduces the mechanical strength of the membrane. Thus, the establishment of high ion conductivity at low water uptake is highly pursued in the synthesis of ion exchange membranes in electrochemical applications, including fuel cells. The water uptake depends on the polymer backbone structure as well as the number and type of active functional groups attached on the polymer backbone. As shown in Figure 2a, the lowest water uptake of 28.44% at 30 °C is displayed by XPAES-DP membrane because of the internal cross-linking via the formation of covalent bonds between the polymer chains. This network structure prohibits the accommodation of a large amount of water molecules and thus results in relatively low water uptake without significant loss of mechanical strength of the membrane. The highest water uptake 34.65% at 30 °C and 56.42% at 80 °C are observed from PAES-P membrane. The higher water behavior of PAES-P than PAES-TM is caused by the hydrophilicity difference between the two functional groups, P and TM. The same trend is observed for the swelling ratio of the membranes as depicted in Figure 2b. Even though XPAES-DP has more quaternary ammonium groups than PAES-TM and PAES-P membranes, its swelling ratio is the lowest because cross-linked molecular structures tend to restrict the expansion of polymer volume. This dimensional stability associated with the low swelling ratio is another advantageous membrane property directly affecting the long-term based cell performance.

### 2.3. Anion Conductivity

**Figure 3:**
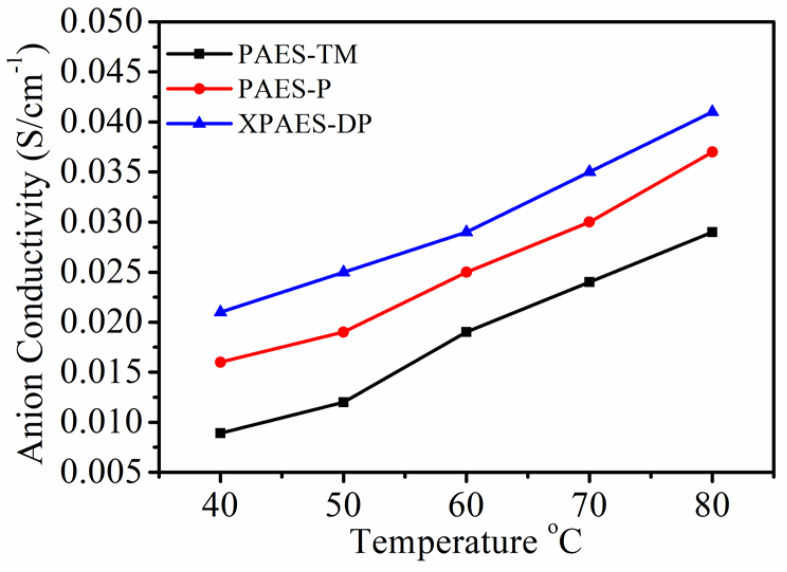
Anion conductivity as a function of temperature for different membranes.

The hydroxide ion conductivity of the synthesized membranes under 100% relative humidity is presented as a function of temperature in Figure 3. For all membrane samples, the conductivity increased with the temperature because of the enhancement of kinetic motion of the anion under more water uptake at a higher temperature. The highest conductivity was shown by XPAES-DP membrane at all temperatures. Quite high anionic conductivity of 0.023 S cm^−1^ was achieved at quite low water uptake of 28.44% at 40 °C. It can be stated that the mobility of the hydroxide ions becomes enhanced in the presence of more quaternary ammonium groups acting as anion carriers for anion hopping. Moreover, the ionic clusters formed by the branched cross-linked structure in the polymer backbone could lead to the formation of ionic channels for facile anion transport by vehicular mechanism. The lower basicity of cyclic-alkyl rings than the conjugated ones would also be another factor for enhanced anion conductivity. The presence of delocalized π-electrons in aromatic ring tends to increase the basicity of the system so that the anions may suffer from the repulsive forces in their percolated path.

### 2.4. Chemical and Oxidative Stability of Membrane

**Figure 4:**
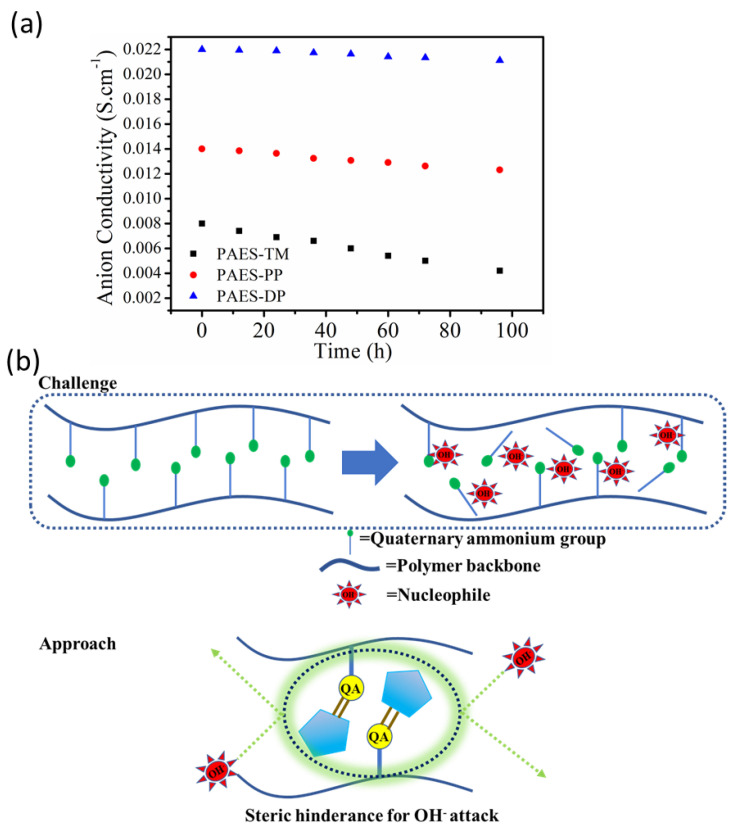
(**a**). Anion conductivity of the membrane as a function of time in alkaline solution and (**b**) the possible mechanism associated with the steric hindrance effect against nucleophiles by the bulky functional groups.

One of the most crucial aspects for the long term application of fuel cell membranes is chemical (alkaline) stability, as the anion exchange membrane fuel cell is operated in alkaline solution. For this evaluation, the membrane samples were dipped in a 3 M alkaline solution for 96 h, to track the variation of anion conductivity. As shown in Figure 4a, the hydroxide ion conductivity remains consistent with a minor change with time for XPAES-DP and PAES-P membrane, while it rather significantly diminishes for PAES-TM. It can be stated that the quaternary ammonium groups attached to the aliphatic side chain are much more susceptible to nucleophilic attack. In this work, the degradation of functional groups is lessened by the following strategies: Introduction of bulkier chain substituted quaternary ammonium group to increase the steric hindrance against hydroxide ions; formation of cross-linking structure in the polymer backbone to provide durability retaining its morphology even after alkali treatment. The possible mechanism associated with the steric hindrance effect against nucleophiles by the bulky functional groups is presented in Figure 4b. In association with this, the highest residual weight was also observed for the crosslinked system after Fenton’s test. The residual weight of XPAES-DP was 89%, which was higher than that of PAES-P and PAES-TM, 82 and 78%, respectively.

### 2.5. Thermal and Mechanical Properties

**Figure 5:**
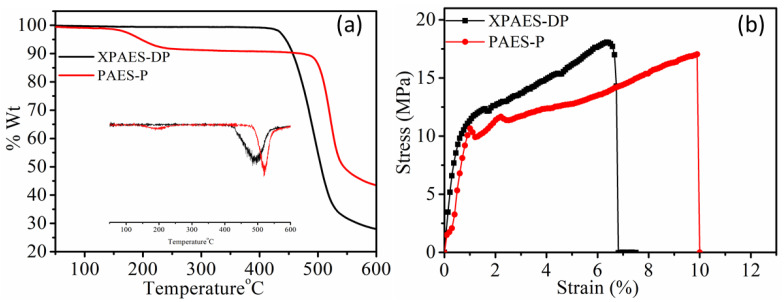
(**a**) Thermal degradation (dTg in inset) and (**b**) tensile stress vs. strain behavior for XPAES-DP and PAES-P membranes.

The temperature-dependent weight loss was observed by TGA for XPAES-PP and PAES-P membranes. The thermal decomposition behavior of the two membranes was slightly different from each other as shown in Figure 5a. The cross-linked XPAES-DP membrane was thermally more stable than the linear PAES-P. While the PAES-P membrane shows the first weight loss at 170 °C associated with the decomposition of functional groups along with the evolution of a slight amount of water. There is slightly less weight loss in the case of XPAES-DP because the cross-linked structure has less tendency to cause decomposition of functional groups as these are covalently bonded with polymer backbone, resulting in a compact and network structure as seen in Figure 5a (inset). The last weight loss of XPAES-DP occurs at 400 °C because of the decomposition of the cross-linked functional groups, followed by the PAES backbone. On the other hand, PAES-P shows a higher decomposition temperature of around 490 °C because its functional groups were already decomposed by the degradation of the polymer backbone.

The mechanical stability was evaluated by UTM for all membranes in the hydrated state as shown in Figure 5b. The cross-linked system, XPAES-DP, shows the highest tensile strength of 18.11 MPa and lowest elongation of breakage of 6.34% as the formation of the covalent bond between the polymer chain leads to a tighter molecular network structure. The low water uptake of this cross-linked membrane is another reason for such a high tensile strength, as the water molecules present in the membrane act as a plasticizer to make the membrane softer. In comparison between the two linear PAES with different functional groups, PAES-P showed higher tensile strength as the large-sized cyclic ring (PP) provides more rigidity than the aliphatic chain, while this PAES-P membrane shows the tensile strength of 17.09 MPa and elongation at break is 9.92%.

### 2.6. Cell Performance Test

**Figure 6:**
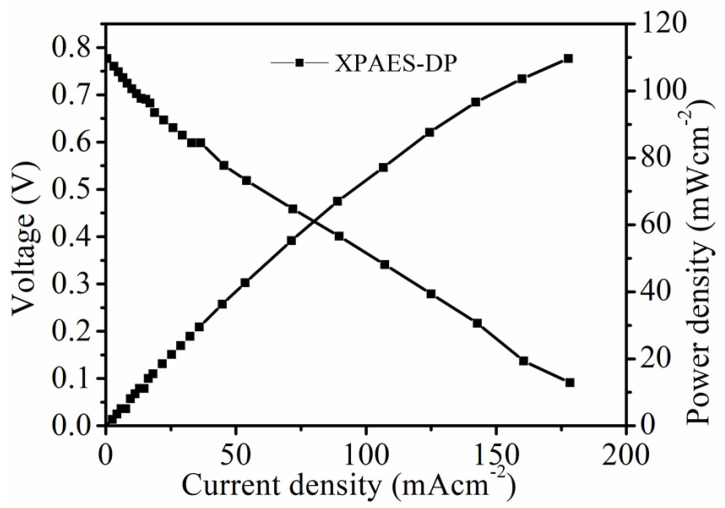
Polarization curve of XPAES-DP at 80 °C and 100 RH.

The single-cell test was performed at 80 °C under 100% relative humidity for the XPAES-DP membrane. The open circuit voltage was 0.78 V and the maximum power density was109 mWcm^−2^, respectively, as shown in Figure 6. As, the quaternary ammonium groups attached on the crosslinked chains act as a chemically stable medium for anion transportation during cell test, the cell displays a very slow rate of voltage drop with increasing current density.

## 3. Materials and Methods

### 3.1. Materials

N-methylpiperidine (99%), 4,4′ -trimethylenebis(1-methylpiperidine), 2,2-bis(4-hydroxy-3-methylphenyl) propane (BHMPP), M_w_ = 85,000 and M_w_/M_n_ = 2.199), were obtained from Sigma-Aldrich (St. Louis, MO, USA). Bisphenol A (BPA), bis(4-fluorophenyl) sulfone (>99%, BFPS), 1,1,2,2-tetrachloroethane (>97%, TCE), potassium carbonate (K_2_CO_3_), benzoyl peroxide (BPO, wetted with 25% water), N-bromosuccinimide (NBS, 98%) and dimethyl sulfoxide (DMSO) were purchased from TCI (Tokyo, Japan) and chloroform (CHCl_3_), isopropyl alcohol (IPA), and deionized (DI) water were supplied from Daejung chemicals (Siheung, Korea). Platinum (nominally 40% on carbon black, HiSPEC 4000) was purchased from Alfa Aesar (Ward Hill, MA, USA).

### 3.2. Synthesis of Methylated PAES (PAES-Me)

Condensation polymerization was carried out form the monomers possessing phenol, fluoride benzene and methylated benzene groups to synthesize PAES-Me [28]. Briefly, BHMPP (7 mmol), BPA (3mmol) and K_2_CO_3_ (2.4 mmol) were mixed to a reaction flask containing a mixture of DMSO and toluene (40:60 in weight) equipped with a dean stark apparatus to remove the byproduct of water. After complete dissolution, the temperature was raised to 150 °C for the polymerization reaction for 4 h. When it was subjected to cooling at 50 °C, 1 mmol of BFPS was immediately added under stirring for 2 h. In the next step, the reaction mixture was subjected for 72 h at 155 °C to carry out the complete condensation polymerization. The phase separated grey colored viscous solution at the bottom of flask was the polymer product. It was centrifuged after dissolution in THF to remove the salt (KCl) and then precipitated in 1000 mL of IPA. The white precipitate was dried at 80 °C under vacuum to obtain the final product PAES-Me. Its synthetic route and chemical structure are shown above in Scheme 1.

### 3.3. Synthesis of Bromomethylated PAES (PAES-MeBr)

A free-radical substitution reaction with BPO and NBS was carried out to synthesize PAES-MeBr [28]. In detail, 1 mmol of PAES-Me was dissolved in 150 mL tetrachloroethane in a round bottom flask containing a magnetic stirrer. After complete dissolution, 20 mmol of NBS and 2 mmol of BPO were added to the reaction mixture. The whole reaction was carried out for 24 h at 65 °C. Afterward, the temperature was cooled down to room temperature and the product was precipitated in 1000 mL of IPA. The product was washed several times with DI water to remove impurities and then dried in a vacuum oven for 24 h at 80 °C. The obtained product was designated as PAES-MeBr as shown above in Scheme 1.

### 3.4. Synthesis of Dual Quaternized Cross-Linked PAES (XPAES-DP)

A predetermined amount of PAES-MeBr was completely dissolved in DMSO at 55 °C to prepare 10 wt.% solution. 4,4′-Trimethylenebis(1-methylpiperidine), 0.25g was added to the resulting polymer solution and kept for 4 h at the same temperature. After this, the solution was cast on a clean glass plate and dried in an oven for 24 h at 80 °C. After the complete drying, the membrane was peeled off and dipped into 0.1 M NaOH solution for complete ion exchange. The two other membranes taken into comparison were quaternized with trimethyl amine and N-methyl piperidinium, respectively, following the same procedure mentioned above. The three synthesized membranes are designated as XPAES-DP, PAES-TM, and PAES-P, respectively, depending on their structure and quaternizing agent.

### 3.5. Characterization

#### 3.5.1. Chemical Structure Analysis

Fourier–Transform infrared spectroscopy (FT-IR) spectra were measured using a Perkin-Elmer FT-IR Frontier instrument (Nicolet iS10, Thermo Fisher, MA, USA). Bromomethylation and sulfonation of polymer were characterized by ^1^H-NMR by 500MHz nuclear magnetic resonance spectrometer (^1^H NMR, Varian Unity INOVA 500MHz, Varian, Palo, Alto, CA, USA) using CDCl_3_ as solvent.

#### 3.5.2. Anion Conductivity

The membrane samples with 3 cm (length) × 1 cm (width) × ~80 μm (thickness) dimension were immersed in water. The hydrated sample was placed in the 4-probe cell (BEKKTECH, LLC, Loveland, CO, USA) to measure anion conductivity by alternating current (AC) impedance spectroscopy in the frequency range from 1 Hz to 1 MHz at 5 mV under 100% relative humidity. The bulk resistance of the membrane was directly obtained from the impedance curve and the anion conductivity of the membrane was determined from the resistance using Equation (1):

(1) $$\sigma =\frac{L}{R\;W\;T}$$

Here, σ is the hydroxide ion conductivity of the membrane in (S cm^−1^), L is the distance in the direction of the ion flow between the measurement probes in cm, R is the bulk resistance of the membrane in ohm, W is the width of the membrane in cm, and T is the thickness of the membrane in cm.

#### 3.5.3. Thermal and Mechanical Properties

Thermal degradation behavior of the synthesized membranes was examined using the thermogravimetric analysis (TGA, Seiko Exstar 6000, Chiba, Japan) with a scan rate of 10 °C min^−1^ from 25 °C to 600 °C under N_2_ atmosphere. The mechanical property of the membrane was analyzed employing the universal testing machine (UTM, Model 5565, Lloyd, Fareham, Hampshire, UK) under a load cell of 250 N. The sample dimension was 4 cm × 1 cm.

#### 3.5.4. Water Uptake and Swelling Ratio

Water uptake is important for the anion exchange membrane because the ion transportation mostly takes place through the bound water inside the membrane via hoping and vehicular mechanisms.

The water uptake was calculated from the following Equation (2).

(2) $$WU\left(\%\right)=\frac{W_{w}-D_{w}}{D_{w}}\ldots$$

where, *W_w_* is the weight of wet membrane and *D_w_* is the weight of dry membrane, respectively. Here, the membrane sample was dipped into deionized water upto equilibrium. After this, the mambrane was wiped with clean tissues to remove tiny water droplets accumulated on its surface to measure its wet weight (*W_w_*). This membrane was placed into an oven at 80 °C for at least 24 h to completely remove the moisture for the calculation of dry weight. 

Moreover, the swelling ratio was calculated from Equation (3):

(3) $$\mathrm{Swelling}\;\mathrm{ratio}=\frac{L_{s}-L_{d}}{L_{d}}$$

where *L_s_* is the length of the wet sample and *L_d_* is the length of the dry sample, respectively. The wet and dry lengths of the sample were measured following the same method.

#### 3.5.5. Ion Exchange Capacity (IEC)

The IEC of the membranes was calculated by Mohr’s titration method. The membrane samples were washed with DI water and completely dried to measure its weight (in gram) before immersion in 0.1 M NaCl solution. The membrane samples charged with Cl^−^ ions were further immersed into 0.5 M Na_2_SO_4_ solution for complete exchange of Cl^−^ ion into SO_4_^2−^ ions. The released chloride ion was titrated against 0.1 M AgNO_3_ using potassium chromate as an indicator. The IEC (meq g^−1^) values of membranes were calculated using Equation (4):

(4) $$\mathrm{IEC}=\frac{C_{\mathrm{Cl}}\times V_{\mathrm{sol}}}{W_{dry}}$$

where *C_cl^−^_* is the concentration of *Cl*^−^ in the extraction solution, *V_sol_* is the volume of titrated or consumed AgNO_3_ and *W_dry_* is the dry membrane weight.

#### 3.5.6. Chemical Stability

The PAES-x sample pieces with 3 cm × 1 cm dimension were immersed in 3 M KOH solution for 96 h at room temperature. Each sample was taken out of the solution to be washed with water repeatedly. After a regular interval of time, the ionic conductivity was measured to justify the chemical stability of the membranes.

#### 3.5.7. Oxidative Stability

Oxidative stability of synthesized different membranes was investigated by assessing the residual weight percentage of each after Fenton’s solution treatment at 60 °C. The rectangular membrane pieces were immersed in the Fenton’s solution (3 wt.% H_2_O_2_, 4 ppm Fe^2+^) at 80 °C for 8 h. After completion of time, the sample pieces were washed several times with DI water and then dried at 80 °C. The residual weight percentage (RW) was calculated by the difference in the weight of the sample before (m_b_) and after treatment (m_a_) from Equation (5).

(5) $$\mathrm{RW}\left(\%\right)=\frac{m_{a}}{m_{b}}\times 100$$

#### 3.5.8. MEA Fabrication and Fuel Cell Test

The catalyst ink was prepared by dispersing 0.1 g of Pt/C (40%) in 0.6 g of Nafion ionomer (5 wt.% in IPA). Additionally, 1 mL of DI water with 8 g of IPA were added to the mixture of Pt/c and Nafion. After sonicated with the help of horn type sonicator (Sonomasher, SL Science, Seoul, Korea) for 30 min, the mixture was sprayed onto the carbon paper to prepare a gas diffusion layer (GDL). After this, the membrane electrodes assembly (MEA) was prepared by pressing the catalyst coated membrane using a heating press (Ocean Science, Seoul, Korea) at 110 °C and 5 MPa for 3 min. The active area of the MEA for this process was 6.25 cm^2^ and Pt loading amount for anode and cathode was 0.8 mg cm^−2^ each. The fuel cell performance was measured using a unit cell station (SPPSN-300) provided by CNL Energy (Seoul, Korea). During the cell test, hydrogen and oxygen gas was continuously fed to anode and cathode sites at the flow rate of 300 cm^3^ per min, respectively. The fuel cell performance was measured at 80 °C under 100% relative humidity (RH).

## 4. Conclusions

A successful approach towards the simultaneous enhancement of anion exchange membrane properties including ion conductivity, mechanical, dimensional, and chemical stability has been carried out by grafting dual quaternized piperidinium groups on the cross-linked PAES. The successful synthesis of XPAES-DP, PAES-P, and PAES-TM was assured by ^1^H-NMR and FT-IR analysis. The excellent thermal stability up to 400 °C and tensile strength of 18.11 MPa are shown by XPAES-DP membrane which were significantly higher than those of PAES-P. Among three membranes, XPAES-DP membrane showed the highest anion conductivity of 0.021 S cm^−1^ at the lowest water uptake of 28.44% because of the presence of a greater number of active functional groups in the cross-linked backbone structure. The chemical stability test also demonstrated that the cross-linked polymer membrane, XPAES-DP, showed much better stability than the linear polymer membranes, PAES-P and PAES-TM, because of the creation of steric hindrance of large cyclic rings grafted on the chemically invulnerable cross-linked backbone structure. Furthermore, when a single cell est was analyzed for the synthesized cross-linked membrane, it displayed the power density of 109 mWcm^−2^ at 80 °C and 100% (RH) condition. All the above results revealed that the synthesized cross-linked membrane bearing dual quaternary ammonium groups could be promising candidates for an anion exchange membrane fuel cell.
